# Assessing Team Performance in a Longitudinal Neonatal Resuscitation Simulation Training Program: Comparing Validity Evidence to Select the Best Tool

**DOI:** 10.7759/cureus.68810

**Published:** 2024-09-06

**Authors:** Sierra Soghikian, Micheline Chipman, Jeffrey Holmes, Aaron W Calhoun, Leah A Mallory

**Affiliations:** 1 Maine Track Program, Tufts University School of Medicine, Boston, USA; 2 Medical Education and Simulation, Hannaford Center for Safety, Innovation and Simulation, MaineHealth Brighton Campus, Portland, USA; 3 Emergency Medicine, MaineHealth Maine Medical Center, Portland, USA; 4 Pediatrics and Critical Care Medicine, University of Louisville, Louisville, USA; 5 Medical Education and Simulation, Hannaford Center for Safety Innovation and Simulation, MaineHealth Brighton Campus, Portland, USA; 6 Pediatric Hospital Medicine, MaineHealth Barbara Bush Children's Hospital, Portland, USA

**Keywords:** assessment tools, healthcare simulation, neonatal resuscitation program (nrp), team performance, validity assessment

## Abstract

Introduction

Neonatal resuscitation is a high-acuity, low-occurrence event that requires ongoing practice by interprofessional teams to maintain proficiency. Simulation provides an ideal platform for team training and evaluation of team performance. Our simulation center supports a longitudinal in situ simulation training program for delivery room teams. In addition to adherence to the Neonatal Resuscitation Program standards, team performance assessment is an essential component of program evaluation and participant feedback. Multiple published teamwork assessment tools exist. Our objective was to select the tool with the best validity evidence for our program’s needs.

Methods

We used Messick’s framework to assess the validity of evidence for potential teamwork assessment tools. Four possible tools were identified from the literature: the Mayo High Performance Teamwork Scale (Mayo), Team Performance Observation Tool (TPOT), Clinical Teamwork Scale (CTS), and Team Emergency Assessment Measure (TEAM). Relevant context included team versus individual focus, external evaluator versus self-evaluation, and ease of use (which included efficiency, clarity of interpretation, and overall assessment). Three simulation experts identified consensus anchors for each tool and independently reviewed and scored 10 pre-recorded neonatal resuscitation simulations. Raters assigned each tool a rating according to efficiency, ease of interpretation, and completeness of teamwork assessment. Interrater reliability (IRR) was calculated using intraclass correlation for each tool across the three raters. Average team performance scores for each tool were correlated with neonatal resuscitation adherence scores for each video using Spearman’s rank coefficient.

Results

There was a range of IRR between the tools, with Mayo having the best (single 0.55 and multi 0.78). Each of the three raters ranked Mayo optimally in terms of efficiency (mean 4.66 + 0.577) and ease of use (4+1). However, TPOT and CTS scored highest (mean 4.66 ± 0.577) for overall completeness of teamwork assessment. There was no significant correlation to NRP adherence scores for any teamwork tool.

Conclusion

Of the four tools assessed, Mayo demonstrated moderate IRR and scored highest for its ease of use and efficiency, though not completeness of assessment. The remaining three tools had poor IRR, which is not an uncommon problem with teamwork assessment tools. Our process emphasizes the fact that assessment tool validity is contextual. Factors such as a relatively narrow (and high) performance distribution and clinical context may have contributed to reliability challenges for tools that offered a more complete teamwork assessment.

## Introduction

Safe healthcare relies on effective collaboration by interprofessional (IP) teams, each sharing expertise to holistically care for a patient. To support this goal, the Institute of Medicine has called for an overhaul of health profession education to emphasize IP teamwork [1]. Simulation offers an ideal setting to practice teamwork skills in a realistic but controlled setting. To effectively utilize simulation training for this purpose, objective and standardized assessment methods are needed to evaluate performance. Multiple teamwork assessment tools with varying degrees of validity evidence have been published for the assessment of individual and overall team performance in a simulated setting. The plethora of teamwork tools makes it difficult to select the single optimal tool for any training program [2-5].

Neonatal resuscitation is a high acuity but low occurrence event, required, on average, in 2% of live births [6]. Successful neonatal resuscitation requires effective teamwork to rapidly implement an evidence-based series of interventions. As such, it is well suited for simulation-based training, which offers regular opportunities for realistic deliberate practice. Many studies describe successful simulation-based neonatal resuscitation training programs [7,8], and some evaluate team performance as part of programmatic outcome evaluation [9,10]. While numerous systematic reviews have recently been published comparing the validity evidence of different teamwork assessment tools in various contexts, none have specifically addressed the unique challenges of neonatal resuscitation training.

Our simulation center conducts ongoing interprofessional team training programs in various settings, including a longitudinal tele-simulation neonatal resuscitation program (NRP) implemented in hospitals across our rural health network. To evaluate this program, we use a scoring tool to evaluate procedural adherence to NRP and another to assess team performance [11,12]. As we expand this NRP tele-simulation training program across eight rural hospitals in our health network, generating up to 40 simulated scenarios per year for evaluation, the need for a single, optimal teamwork tool becomes increasingly apparent. This project aims to compare the validity of four candidate team performance assessment tools and select the most suitable one to provide formative participant feedback and to evaluate our simulation-based team training program.

This work was previously published as a meeting abstract at the International Pediatric Simulation Symposia and Workshop (IPSSW) on May 7, 2024, and at the Costas Lambrew Research Retreat on May 1, 2024.

## Materials and methods

Context 

The Maine Ongoing Outreach Simulation Education (MOOSE) program uses tele-simulation to practice and improve neonatal resuscitation, communication, and teamwork across our rural hospital system. The MaineHealth Institutional Review Board determined the MOOSE program was exempt from full review (approval number: 2050668-3).

Interprofessional delivery room teams gathered monthly for one year and then quarterly thereafter to conduct a single NRP mega-code in situ. The simulation was produced by on-site educators and was observed by neonatal simulation experts remotely from Maine Medical Center, Portland, Maine, United States, the tertiary care hospital where the Level 3 neonatal intensive care unit (NICU) and simulation center are located. One neonatologist and one simulation educator facilitated a reflective debrief over Zoom (Zoom Video Communications, Inc., San Jose, California, United States) following each simulated scenario.

To launch this program, we conducted a one-year pilot in a single rural hospital [12]. To assess team performance during this pilot, we reviewed the previously recorded trainings, and then two simulation educators used the Mayo High-Performance Teamwork Scale (Mayo) [13] and the Team Strategies and Tools to Enhance Performance and Patient Safety (TeamSTEPPS™), Team Performance Observation Tool (TPOT) [14,15] to assess and track team performance for each session. In this pilot, teams demonstrated improvement according to both scales [11]. As we disseminate the training program across seven more rural hospitals, we aim to identify a single team performance assessment tool for our program to improve efficiency and decrease workload.

Subject demographics

Participants across the 10 sessions included 11 physicians, 27 nurses, and nine respiratory therapists (RT) for 47 participants in total. Each simulation had between four and six participants, all had at least one physician, and all but one had one RT. The 10 sessions included four different rural hospitals from our system. The hospitals’ annual birth rates ranged from 100-230 live births per year. Two are designated as critical access hospitals. None have pediatricians or anesthesiologists staffed in-house 24/7 (overnight call from home).

Raters included three health professionals, each with >10 years of experience facilitating simulation-based interprofessional team training in multiple disciplines. LM is a pediatric hospital medicine physician and is Director of the Simulation Center, MC is a critical care nurse and a clinical educator at the Simulation Center, JH is an emergency medicine physician and Regional Director of Simulation.

Tool comparison

We used Messick’s framework to ground our assessment of validity evidence for candidate tools for the purpose of providing formative feedback to participants, and native teams of interprofessional healthcare providers who resuscitate neonates in our rural hospital delivery rooms. This data was also used to evaluate the simulation training program which aims to improve team performance through ongoing simulation training. Data provided by this assessment tool may be used to modify content in simulation debriefing to improve team performance [16].

Content Evidence

We conducted a literature search to identify assessment tools with validity evidence for our context, including relevant systematic reviews. Contextual elements relevant to our program included design for use by an external rater measuring interprofessional team performance in a simulated setting. Additionally, in our rural context, neonatal resuscitation is managed by relatively small teams (typically four individuals). Resuscitation is usually initially managed by two labor and delivery nurses with a respiratory therapist called, as well as a provider arriving from home or clinic. These expected transitions in team composition and often leadership make a clear identification of team leader, roles and responsibilities, and hand-off communication especially important. For this reason, teamwork constructs were identified as important to our team as we considered tools that included leadership, roles and responsibilities, and communication.

A literature search yielded several recently published systematic reviews evaluating validity evidence for team performance tools for interprofessional simulations [5], obstetric emergencies [2,3], trauma teams [17], and crisis situations [18]. Wooding et al. summarized 24 scales and found that three (TPOT, Mayo, and the Team Emergency Assessment Measure (TEAM)) emerged with “good validity evidence underpinning their use” [5]. Boet et al. included 13 scales and concluded that TEAM “may be the most promising given its measurement evidence” [18]. Bhangu et al.’s trauma-focused review also concluded that TEAM had a higher level of evidence. Both obstetric-focused reviews found the Clinical Teamwork Scale (CTS) [19] to have the most comprehensive evaluation [2,3]. Four tools (Mayo, TPOT, CTS, and TEAM) emerged with more robust published validity evidence. They seemed sufficiently feasible (length and ease of interpretation) according to our program needs and were selected for further validity analysis. Tool characteristics and available validity evidence are summarized in Table 1.

**Table 1:** Description of each candidate tool including validity data from initial publication TeamSTEPPS: Team Strategies and Tools to Enhance Performance and Patient Safety

Teamwork Assessment Tool	Tool description	Validity	Notes
Mayo High Performance Teamwork Scale (Mayo)	Tool contains 16 classifications of teamwork to produce a total score, uses three-point Likert scale	Internal consistency and construct validity by Rasch (person reliability = 0.77; person separation = 1.85; item reliability = 0.96; item separation = 5.04) and traditional psychometric (Cronbach's alpha = 0.85) [13]. Additional validity data from subsequent publication [20]	Items 9-16 conditional (may not be relevant in every scenario)
Team Strategies and Tools to Enhance Performance and Patient Safety (TPOTS)	Tool contains 22 total items divided into five categories: team structure communication, leadership, situation monitoring, mutual support that uses five-point Likert scale	Validity data from subsequent publication that created anchors to improve reliability yielded test-retest reliability (kappa = 0.707, p < 0.001), interrater reliability (kappa = 0.730), and good internal consistency reliability (Cronbach's α = 0.921) [15]	Designed to be used within TeamSTEPPS program
Clinical Teamwork Scale (CTS)	Tool contains 16 items in five conceptual teamwork domains, each with sub-items and its own overall score: communication, situational awareness/resource management, decision-making, role responsibility (leader/helper), and patient-friendliness. One item is a yes/no (target fixation). Subsequent items use a 0-10 Likert scale. Final item is a 0-10 global rating scale	Agreement (Kappa 0.78) and score concordance (Kendall coefficient 0.95) among three raters, and interrater reliability (interclass correlation coefficient 0.98) [19]; Additional validity data from subsequent publication [2]	From standardized videos using actors representing one of three team performance ratings (Unacceptable, average, and good)
Team Emergency Assessment Measure (TEAM)	Tool contains 12 items in three categories: leadership, teamwork, and task management. Uses a 0-4 Likert Scale for items 1-11. Final item is a 0-10 global rating scale	Correlation between the total item score and global rating (rho 0.95; p<0.01) indicated concurrent validity. Inter-rater (k 0.55) and test-retest reliability (k 0.53) [21]. Additional validity data (simulated setting) from subsequent publication [10,22] including recorded clinical encounters [23]	Some “rating prompts” included for items within scale

Response Process

We sought to assess the raters’ experience with the tools. Three simulation experts (LM, MC, JH) began by reviewing a single pre-recorded video of a neonatal resuscitation from the MOOSE program and individually assessed teamwork using the four-team performance tools. Ratings were then reviewed by the group and each item discussed to reconcile interpretation and identify exemplars by consensus for tool items where the raters noted disparate interpretation.

Each rater then independently rated five MOOSE sessions using the four tools. Analysis of rater reliability (see Internal Structure below) at that point revealed poor reliability for several scales, so raters again reviewed and reconciled an additional session in an attempt to improve agreement. Raters then went on to independently rate five additional sessions. Videos were selected randomly, chronologically, using every other session.

After evaluating the 10 videos, each rater answered three questions designed by the research team to evaluate the rater experience with each tool using a five-point Likert scale (1= strongly disagree to 5= strongly agree): (i) The tool was efficient to complete/took a reasonable amount of time; (ii) The items were clear and easy to interpret; (iii) The scale provides a complete assessment of teamwork.

Internal Structure

We calculated interrater reliability (IRR) for each of the candidate tools across the 10 scored sessions using intraclass correlation (two-way random, absolute value, single and multiple raters) for each tool across the three raters.

Relationship to Other Variables

To assess the relationship to other variables, we compared average total team performance scores for each tool to NRP adherence scores obtained as part of the training program [12]. Spearman’s Rank Coefficient was used given the non-parametric distribution of data.

All statistics were performed using IBM SPSS Statistics for Windows, Version 29.0 (Released 2023; IBM Corp., Armonk, New York, United States).

## Results

The three raters ranked Mayo and TEAM most favorably in terms of efficiency (mean 4.66 ± 0.577) and ease of use (4 ± 1/0). Rater perceived ‘completeness’ of a tool to team performance for Mayo was lower (3.66 ± 0.577) compared to the other three teamwork tools assessed. For this dimension, TPOT and CTS were rated highest (mean 4.66 ± 0.577) (Table 2).

**Table 2:** Rater interpretation of teamwork tools using a five-point Likert scale

Teamwork Tool	Efficient to complete, reasonable amount of time, mean ±SD	Items clear and easy to interpret, mean ±SD	Scale provides a complete assessment of teamwork, mean ±SD	Total, , mean ±SD
Mayo High-Performance Teamwork Scale (Mayo)	4.66 ± 0.577	4 ± 1	3.66 ± 0.577	12.33 ± 0.508
Team Strategies and Tools to Enhance Performance and Patient Safety (TPOTS)	3 ± 1	2.66 ± 0.577	4.66 ± 0.577	10.34 ± 1.07
Clinical Teamwork Scale (CTS)	4.33 ± 0.577	3.33 ± 0.577	4.66 ± 0.577	12.32 ± 0.69
Team Emergency Assessment Measure (TEAM)	4.66 ± 0.577	4 ± 0	4 ± 1	12.66 ± 0.38

The four teamwork tools assessed had a range of IRR. Mayo had the best ICC, single 0.55 and multi 0.78 (Table 3). ICC for TPOT was 0.21 single and 0.44 multi, for CTS it was 0.07 single and 0.19 multi, and for TEAM it was -0.08 single and -0.27 multi. 

**Table 3:** Interrater reliability of the four candidate tools based on rating of 10 videos by the three raters

Teamwork Tool	Clinical Teamwork Scale (CTS)	Team Strategies and Tools to Enhance Performance and Patient Safety (TPOTS)	Team Emergency Assessment Measure (TEAM)	Mayo High Performance Teamwork Scale (Mayo)
Interrater Reliability Single	0.07	0.21	-0.08	0.55
Interrater Reliability Multi	0.19	0.44	-0.27	0.78

Relationship to other variables

Comparing the average teamwork assessment rating for each tool to the overall NRP adherence score, Spearman rank coefficient values varied from 0.01 (p=0.99) for CTS to 0.47 (p=0.17) for Mayo. For TPOT, it was 0.44 (p=0.2) and for TEAM, it was 0.04 (p-0.91). No correlation was significant.

## Discussion

Assessment of teamwork is necessary to fully implement simulation-based team training programs. These initiatives are commonly used to maintain readiness for high-acuity, low-occurrence emergencies that include neonatal resuscitation. This study is the first, to the best of our knowledge, to compare the validity of several available teamwork tools for use with an NRP simulation training program. We narrowed our consideration to four candidate tools based on previously published validity evidence for teamwork assessment tools. We then analyzed IRR amongst three raters for 10 scenarios from our program, the rater evaluation of each tool, and the relationship of each score to NRP adherence for our context. Of the tools we considered, Mayo emerged as the most suitable tool for our purposes. However, team performance scores did not correlate with technical (NRP adherence) performance for any tool, including Mayo, and IRR for three of the four tools was surprisingly poor. For Mayo, IRR was moderate and we were unable to conclude that we had sufficient rater reliability to proceed with single rater analysis as was our original intent.

As interprofessional teams are increasingly identified as the unit of care delivery and high-quality teamwork a critical aspect of patient safety, many simulation training programs seek to assess team performance. Published works comparing different team performance assessment tools for simulation training programs report widely variable validity data, including rater reliability [2-5,18]. Several reviews found that the TEAM tool was associated with stronger validity evidence than others [4,18]. In our study, TEAM received the highest overall rating from assessors supporting the response process element of validity but proved problematic in terms of IRR. McKay and colleagues concluded that TEAM demonstrated excellent IRR between two experienced faculty raters for adult resuscitation simulations [24]. However, they analyzed each element of the TEAM scale items individually, and half of the 12 items demonstrated statistically significant disagreement. They did not evaluate rater reliability for overall scores, the primary outcome unit used for our program evaluation. 

Several factors could contribute to our challenges in achieving higher rater reliability for TEAM, CTS, TPOT, and, to a lesser extent, Mayo. Non-technical components of teamwork are inherently subjective, making IRR challenging. Several other published reports demonstrate poor IRR [10,25]. Zhang et al. recognized this and attempted to decrease the subjectivity of the TPOT by adding scenario-specific anchors to the tool [15]. By doing so they achieved an IRR of 0.730. With additional rater training and developing anchors specific to neonatal resuscitation, we could likely improve our IRR. However, we intended to choose a tool that required minimal rater training to support a more sustainable implementation of program evaluation. 

The spectrum of team performance can also impact rater reliability. Some reports of the highest IRR utilized simulations with the same actors deliberately portraying very good, medium, and sub-optimal team performance across the same scenario in a simulation laboratory to assess tool validity [19]. In our context, teams comprised practicing providers in their native roles and settings. Reassuringly, performance over the 10 scenarios was distributed across a relatively narrow performance spectrum, in the good to very good range. For instance, 25 of 30 global rating scale assignments associated with TEAM (1-10 scale) were scored 7 or 8. This narrow distribution of performance may have made rater reliability harder to achieve.

As validity is context specific, it is possible that the scenario of neonatal resuscitation team performance may be associated with decreased reliability relative to other situations. Rovamo et al. looked at the effect of an intervention on IP teamwork in simulated neonatal scenarios and used the TEAM assessment tool. Interestingly, they also found that the interrater agreement between the three anesthesiologist raters was poor to moderate (Cohen’s kappa 0.2-0.5) [10]. Compared to other resuscitation scenarios (e.g. trauma), NRP is very protocol driven and may present fewer opportunities for disagreement about the correct next steps. This could make it more difficult to discriminate team performance.

Rater background and training may also significantly impact the ability to reliably use tools [26]. Our raters represent different professions and disciplines, and each has over a decade of experience in using simulation for team training. This experience could make rater reliability more difficult to achieve as perspectives may be more entrenched in their opinions and assessments [27].

The lack of correlation in our study between technical team performance (as measured by NRP adherence) and teamwork is interesting. A 2019 meta-analysis of the relationship between teamwork and performance in healthcare teams found an overall “medium-sized” effect of teamwork on performance with an r=0.28 [28]. Only two studies have looked at the relationship between teamwork and neonatal resuscitation, and neither have used standardized tools for the assessment of team performance or NRP adherence. One found that “vigilance” was associated with fewer errors in NRP [29] and the other found that “standardized communication techniques” were not significantly associated with decreased NRP errors [29,30]. This suggests that there may be discrete aspects of teamwork best practices that are associated with NRP technical performance, but with the broader lens we used of a composite score, teamwork does not correlate.

Ultimately, the validity of any assessment tool is unique to the context in which it is applied. The higher the stakes of the assessment, the more crucial the validity argument becomes. In our case, the priority was to evaluate our simulation training program’s efficacy in optimizing team performance and to provide formative feedback to participants. As such, “good” interrater reliability may be acceptable. Additionally, a tradeoff between the best “complete” assessment of teamwork for an efficient and easy-to-interpret tool with satisfactory rater reliability (Mayo), may be warranted. However, it is important to note that even the “best” tool for our purposes fell short of sufficient rater reliability to justify a transition to single rater analysis of each session as was our initial hope to improve program sustainability.

Limitations

While we used existing systematic reviews to select the four most appropriate candidate tools to test for our context, there are undoubtedly other relevant tools that we might have evaluated, and our selection of four is somewhat subjective. We assessed team performance while viewing previously recorded simulations, so as to be able to rewind and review to ensure accuracy. We have not attempted to assess team performance in real time during the tele-simulations. Users wishing to use these tools in real time may not be able to apply our analysis to that context. Additionally, our three identified raters bring their own backgrounds and biases to the assessment of team performance elements. A more robust analysis including raters of different backgrounds and experience levels, as well as a more detailed analysis of individual items for each tool would provide a more complete assessment of validity and might suggest means to improve reliability. However, this was outside the scope of the objective of our project which was to identify a pragmatic tool for an ongoing training program.

## Conclusions

Using a validity framework, we reviewed the many teamwork assessment tools to identify the most suitable for our interprofessional in situ neonatal resuscitation team training program. Pragmatic use (limiting required rater training and efficiency) was a priority for our context where we plan to disseminate a program across a health system and influenced how we weighed the strengths and weaknesses of various tools. Mayo scored the highest of the four candidate tools for ease of use, efficiency, and rater reliability, though not completeness of assessment. There was poor IRR for the remaining three tools, which is not an uncommon problem with teamwork assessment tools. Mayo reliability was moderate for multiple raters, but insufficient to justify use by only one rater. Our process emphasizes the fact that assessment tool validity is contextual. Factors such as relatively narrow (and high) performance distribution and clinical context may have contributed to reliability challenges for tools that offered a more complete assessment of teamwork.

The next steps will be to move forward with our NRP tele-simulation training program, using Mayo to evaluate teamwork over time. As we launch other team-based training programs (such as obstetrical emergencies) across our health system, we will use a similar process to vet teamwork assessment tools for that context, with a distinct group of raters, which may yield a different optimal teamwork assessment tool.
